# HIV Post Exposure Prophylaxis: prospects, opportunities and challenges

**DOI:** 10.1002/jia2.26511

**Published:** 2025-06-26

**Authors:** Julie Fox, Euphemia L. Sibanda, Peter Godfrey‐Faussett

**Affiliations:** ^1^ King's College London London UK; ^2^ Guys and St Thomas's NHS Foundation Trust London UK; ^3^ Centre for Sexual Health and HIV/AIDS Research (CeSHHAR) Harare Zimbabwe; ^4^ Liverpool School of Tropical Medicine Liverpool UK; ^5^ Faculty of Infectious and Tropical Diseases, London School of Hygiene & Tropical Medicine London UK

Despite great improvements over the last decade, HIV incidence remains unacceptably high, with 1.2 million acquisitions globally in 2023, and 450,000 in sub‐Saharan Africa [1]. This is way off the global target of 370,000 new HIV acquisitions. The recorded reductions in new acquisitions are attributed to the successful scale‐up of HIV treatment and several prevention interventions, including pre‐exposure prophylaxis (PrEP) [2]. Implementation and uptake of HIV post‐exposure prophylaxis (PEP—use of antiretroviral medication to prevent HIV acquisition after a potential exposure), however, has been limited, despite it being part of World Health Organization (WHO) guidelines since 2014. In many settings, its use has been limited to occupational and sexual violence exposures, with missed opportunities for HIV prevention. PEP is an effective intervention whose improved scale‐up will be important for driving the attainment of prevention targets. Although no randomized trials were conducted, evidence of efficacy comes from animal studies [3, 4], later reinforced by systematic reviews and meta‐analyses [5]. In humans, evidence of efficacy comes from case series, case‐control studies [6] and systematic reviews that underscore the value of PEP [7]. It is also extrapolated from clinical trials investigating perinatal transmission of HIV [8].

Prior to 2024, WHO guidelines recommended that PEP be available from centralized services which put a strain on health systems and led to delays in accessing PEP. Other barriers included a lack of knowledge on PEP among providers, particularly community‐based providers, and potential beneficiaries of PEP [9]. In light of these barriers, in 2024, WHO issued new guidelines which advocate for community‐based distribution of PEP and through task sharing [9].

To facilitate delayed access, PEP guidelines allow for a window of 72 hours from exposure to the first PEP dose despite limited evidence for its efficacy after 24 hours. For those who access PEP, adherence is commonly sub‐optimal with 36–65% completing the full 28‐day course [10] and uptake most often after the critical 24‐hour window period [11]. In the 2024 guidelines, WHO did not recommend changes to the 28‐day duration or window period from exposure to uptake, but instead focussed on rapid uptake and a decentralization of services to facilitate this and greater uptake in general [9]. There is recognition that some individuals using PEP will have repeated or ongoing exposures to HIV and could, therefore, benefit from transitioning from PEP to PrEP. WHO guidelines also provide guidance for this transition [9].

For sub‐Saharan Africa, data on PEP is limited but, as with the global picture, uptake and availability is generally low. There are no large‐scale demonstration projects and with new prevention drugs in the pipeline, a methodology for evaluating PEP drugs is needed: in the era of PrEP and universal test and treat, efficacy studies are no longer affordable due to large sample sizes required. To realize the maximum impact of PEP, it is important to recognize that product innovations with long‐acting HIV prevention drugs present the possibility of a single drug dose for PEP but as with other areas of medicine, there will be challenges in moving to wide‐scale implementation particularly when drug quantities for PEP are not high volume [12].

For this supplement, we invited the submission of multidisciplinary articles designed to advance the rollout of PEP in sub‐Saharan Africa. After careful review, the editorial team selected 17 contributions that illustrate current PEP advances and challenges to improve the delivery and uptake of PEP across sub‐Saharan Africa.

As new drugs are developed for PEP and PrEP, we need to overcome longstanding challenges to assessing them in the presence of lower HIV incidence and availability and ethical responsibility to providing PrEP in prevention studies. The supplement is divided into five sections, covering the PEP pipeline and challenges for investigating new regimens, WHO PEP guidelines and implications for Africa, and three sections covering different aspects of PEP research from within Africa.

In the first section, we provide two articles, the first discussing trial designs to show the efficacy of PEP (Ortblad et al.) [13] and the second describing regulatory pathways for licensing of new PEP drugs (Miller et al.) [14]. This section is drawn to a close by a pharmacokinetics modelling paper, evaluating the potential efficacy of 2 and 3 drug PEP, with a duration from exposure to dosing and finally duration of dosage (Von Kleist et al.) [15].

The next section discusses the implications and potential challenges that programmes may face in implementing the WHO guidelines. The feasibility and acceptability of the WHO guidelines are highlighted by the systematic review that Kennedy et al. conducted to explore community delivery of PEP and task shifting [16]. Although the evidence was limited, the review generally suggests positive outcomes—feasibility, acceptability and cost‐effectiveness of the approaches. The commentary by Magni et al. brings together perspectives from programme implementers from five African countries [17]. They show that adoption of the guidelines would require programmes to tackle poor knowledge and acceptability of PEP, programmatic readiness including the need for training of providers, and considerations for improving integration between PEP and PrEP. An important question for the implementation of the guidelines is on cost‐effectiveness, which is addressed in a commentary on the economics of HIV prevention, where Garnett and Godfrey‐Faussett explore the plausibility of cost‐effectiveness of PEP in sub‐Saharan Africa [18]. They review studies that suggest that transmission within a partnership is not linear so it can be assumed that if transmission is going to occur, it is most likely in the first few sex acts without a condom and in which the virus is not fully suppressed. They discuss various determinants of cost‐effectiveness and conclude that although PEP is worthwhile in settings of high HIV prevalence and unsuppressed viral load, it is likely to only be cost‐effective when promoted for the first few unprotected (condomless and/or in virally unsuppressed people) sex acts in new partnerships. An analysis of implementation planning for PEP from five sub‐Saharan African countries by Resar et al. showed a shift to expand the use of PEP beyond occupational and sexual violence exposures [19]. These plans were incorporated in national budgets which highlights potential programme readiness to shift to the new WHO guidelines. Taken together, these four studies demonstrate a promising platform for implementing the WHO guidelines while highlighting the hurdles that need to be addressed.

The next three papers go into detail about specific barriers to implementing the guidelines, particularly highlighting the most vulnerable groups that need to be targeted. Laterra et al. showed poor knowledge of PEP among adolescent girls and young women in Eswatini [20]. The lowest knowledge levels were among participants who had not been reached by programmes. Schluck et al. also highlight poor knowledge of PEP among people vulnerable to HIV acquisition in Kenya, with lack of education being a predictor of poor knowledge [21]. Taken together, these two studies highlight the need to develop models that target hard‐to‐reach groups. Analysis of a cohort of PEP users in Malawi (Tweya et al.) showed that about a third had ongoing exposure to HIV, with high rates of seroconversion reported [22]. This emphasizes the importance of integrating PEP with PrEP and facilitating the appropriate transitions.

The rest of the papers provide insights from different PEP delivery models. Three papers explore PEP in the setting of gender‐based violence and showcase the need to improve the rapid uptake of PEP upon presentation. Kanagasabai et al. present data from 14 PEPFAR‐supported countries where PEP was provided to survivors of sexual violence [23]. They found poor completion rates in this group. Duffy et al. provided important qualitative insights from health workers on how the implementation of PEP for individuals who have suffered sexual violence can be optimized [24]. Adewumi et al. highlight the feasibility of offering PEP to survivors of sexual violence at police stations [25].

The final four papers focussed on implementation research on delivery approaches among different groups with high vulnerability to HIV. These tended to be smaller‐scale studies within novel settings. Kuguyo et al. piloted peer‐led delivery of PEP vouchers among students enrolled in colleges in Zimbabwe [26]. They demonstrated the acceptability of PEP among students, with 30% of students who collected PEP vouchers redeeming them. Roche et al. evaluated pharmacy delivery of PEP and PrEP in Kenya, highlighting the high acceptability of the model and acceptable rates of follow‐up HIV testing after the use of PEP [27]. Naik et al. showcased a pharmacy‐led delivery model and online delivery of both PEP and PrEP, highlighting transitions between the two prevention methods [28]. Ayieko et al. [29] describe approaches to PEP delivery within the SEARCH programme. Although participant numbers are still not large, their results highlight that PEP is feasible and an appropriate choice for some people.

Taken together, these studies underscore the importance of acknowledging choice and providing more diverse PEP settings as key tenets to increasing PEP availability and speedy uptake. For PEP to achieve its potential impact on the HIV epidemic, it needs to reach populations with the greatest need. These populations often face disparities in health access, either within countries or regions where health resources are limited.

PEP might substantially reduce HIV in settings where people experience high HIV incidence and for whom PrEP use is not possible, for example cases of gender‐based violence. However, all these papers serve as a reminder that PEP delivery is not easy, evidence for use after 24 hours is not available and uptake dependent on many factors.

Ongoing research and evaluation into number of drugs, duration of therapy and pipeline drugs as well as implementation research to optimize delivery pathways will help inform best practices. This is even more critical in this era of drastic cuts to international funding [30], where modelling has shown that HIV prevention efforts will be the most affected [31].

The future positioning of daily PEP for 28 days is likely to be different given advances in the development of long‐acting prevention agents such as monthly oral MK‐8527 [32]. These long‐acting oral agents are not yet available but are promising advances that can potentially simplify PEP regimens. Providers need to expand PEP user's choice of access methods. HIV prevention in Africa remains crucial if we are to reach the goal of HIV no longer being a public health challenge. Reductions in investment at this stage threaten to reverse a decade of solid progress in the region.

## COMPETING INTERESTS

The authors declare no competing interests.

### AUTHORS’ CONTRIBUTIONS

JF and ELS contributed to the initial draft of the manuscript. PG‐F provided feedback, reviewed and edited the draft, and approved the final version prior to submission.

## FUNDING

The publication of this supplement was supported by the Gates Foundation.

## DISCLAIMER

The authors alone are responsible for the views expressed in this issue. They do not necessarily represent the views, decisions or policies of the institutions with which they are affiliated nor any of the funding agencies supporting their work.

## Data Availability

Data sharing is not applicable to this article as no datasets were generated or analysed during the current study.
